# Multiparametric MRI for Recurrent Prostate Cancer Post Radical Prostatectomy and Postradiation Therapy

**DOI:** 10.1155/2014/316272

**Published:** 2014-05-25

**Authors:** Flavio Barchetti, Valeria Panebianco

**Affiliations:** Department of Radiological Sciences, Oncology and Pathology, Sapienza University of Rome, 00186 Rome, Italy

## Abstract

The clinical suspicion of local recurrence of prostate cancer (PCa) after radical prostatectomy (RP) and after radiation therapy (RT) is based on the onset of biochemical failure. The aim of this paper was to review the current role of multiparametric-MRI (mp-MRI) in the detection of locoregional recurrence. A systematic literature search using the Medline and Cochrane Library databases was performed from January 1995 up to November 2013. Bibliographies of retrieved and review articles were also examined. Only those articles reporting complete data with clinical relevance for the present review were selected. This review article is divided into two major parts: the first one considers the role of mp-MRI in the detection of PCa local recurrence after RP; the second part provides an insight about the impact of mp-MRI in the depiction of locoregional recurrence after RT (interstitial or external beam). Published data indicate an emerging role for mp-MRI in the detection and localization of locally recurrent PCa both after RP and RT which represents an information of paramount importance to perform focal salvage treatments.

## 1. Evidence Acquisition

A systematic review of the literature was performed by searching Medline and Cochrane Library databases from January 1995 up to November 2013 (primary fields: prostate neoplasm and local recurrence after radical prostatectomy, after external beam radiation therapy, and after brachytherapy and MRI). Electronic searches were limited to the English language. Original articles and review articles were included and clinically reviewed. Additional references were identified from reference lists of these articles. We have not included editorials, abstracts, and reports from meetings. We included in the present review only articles specifically and primarily focused on the role of MRI in the detection of local recurrence after radical prostatectomy (RP) and radiation therapy (external beam or interstitial). In our research we found 10 original studies on the use of mp-MRI after RP and 20 studies on the use of MRI focused on the detection of local recurrence after radiation therapy (RT). We principally included in the analysis studies which clearly reported data on local recurrence detection rate, PSA level, number of patients submitted to treatment, and validation criteria for the imaging findings confirmation.

## 2. Background

Prostate cancer (PCa) is second only to lung cancer as a cause of cancer mortality among men in western countries [1]. RP represents the first line treatment with curative intent, followed by RT (interstitial or external-beam) which is becoming a valid alternative to surgery in patients with low- to intermediate-risk PCa and a long life expectancy [2].

Both surgery and RT are definitive treatments for localised PCa and offer long-term tumour control in most patients, but residual or recurrent local disease is a critical issue because it may greatly influence the subsequent therapeutic strategy and patient management.

At present, the diagnosis of local relapse is based mainly on prostate-specific antigen (PSA) level above a threshold or on PSA kinetic values and it is called biochemical failure (BF) or biochemical recurrence (BR) or biochemical progression (BP). However, BF is not synonymous of local recurrence in the prostatic bed. It can be also due to distant metastases, local disease, or both. Moreover, a persistently elevated PSA serum level could be also due to residual glandular healthy tissue in the post-prostatectomy bed.

BR following RP develops in about 50% of high risk patients and in about 10% of low risk patients within 15 years from surgery [30]. The absence of a complete prostatic capsule at the apex and the need to preserve the pelvic structures (essentially urethral sphincter and neurovascular bundles) are the principal reasons of this high incidence of local relapse [31]. As regards RT, BF ranges from 15% for low-risk patients to 67% for high-risk patients during a 5-year period followup [32].

## 3. Clinical Evidence

After RP, serum PSA level should decrease to an undetectable level (<0.1 ng/mL) within 21–30 days and should remain undetectable thereafter. Any detectable level and/or rising PSA after RP should be theoretically considered as persistent or recurrent disease, although extraprostatic PSA production (e.g., produced in epithelial cells of the trachea, thyroid gland, mammary gland, salivary gland, jejunum, ileum, epididymis, epidermis, and pancreas) should be taken into account [33]. According to EAU-guidelines, BF after RP is defined by two consecutive values of serum PSA >0.2 ng/mL [34]. Once that BR occurs, the key question remains whether a PSA rise is reflective of local or distant disease in order to plan the most appropriate treatment. Generally, PSA detectable after 1 year, PSA velocity (PSA_ve_)  <0.75 ng/mL/year, PSA doubling time (PSA_dt_)  >6 months, negative lymph nodes, no seminal vesicle (SV) invasion, positive margins, and Gleason score <7 are all factors related to higher risk of local relapse, while PSA detectable before 1 year, PSA_ve_>0.75 ng/mL/year, PSA_dt_<6 months, positive lymph nodes, SV invasion, and Gleason score >6 are related to systemic relapse [35]. However, in the clinical practice, it is not so easy to identify the origin of the PSA relapse and sometimes many risk factors for both local and distant recurrence are present in the same patient. Moreover it should be taken into account that the PSA level does not always correlate well with the tumour burden and that there are numerous examples of metastatic PCa in the absence of significantly elevated PSA levels, particularly when the tumours are poorly differentiated [36]. Therefore in patients with BF after surgical treatment, a diagnostic imaging procedure is often carried out to distinguish between local cancer recurrence and distant spread of disease. In the absence of systemic metastases salvage RT could theoretically be assumed to be the first line treatment offering a potential chance of cure. However, if systemic disease is diagnosed, RT on the prostate bed would be unnecessary, due to the high risk of morbidity, and the most relevant treatment option is androgen deprivation therapy (ADT) [34]. According to EAU Guidelines salvage radiotherapy with or without ADT should be initiated when PSA levels is <1.0 ng/mL [34]. However, the general feeling is that the lower the PSA level at the time of salvage RT, the better the result and some recent reports suggest that results are best when the serum PSA level is <0.5 ng/mL [37].

Defining BR after definitive RT is a complex issue. The evaluation of serum PSA values after RT has a less defined role than it plays after surgery, as PSA levels decrease slowly and may never reach undetectable levels because varying amounts of PSA producing tissue may remain viable after a curative dose of radiation. According to Phoenix criteria the current standard definition of recurrent and/or persistent disease after RT is a serum PSA level over a threshold of 2 ng/mL above the nadir value [31]. The rate of PSA rise can potentially predict clinical failure patterns: a rapidly rising PSA indicates metastatic recurrence, whereas a moderately rising PSA suggests local relapse [38]. Other studies suggest that patients who develop metastasis have a PSA_dt_ (3 months) and a time to BF (<1 year) significantly shorter than ones who developed a locoregional recurrence [39]. Nevertheless, up to now, no pattern of PSA kinetics after RT has conclusively differentiated between local recurrence and systemic disease [40]. Once BF is established, the next step is the differentiation between local and distant relapse in order to plan the most relevant treatment. In this setting medical imaging plays a crucial role. Salvage therapy offers a potential chance of cure to patients with isolated local recurrence in the prostate gland, while ADT represents the treatment of choice in the presence of systemic disease [41].

## 4. Multiparametric-MRI: Technical Aspects

Multiparametric MRI (mp-MRI) has proven to be the most useful tool available up to now for the detection and localization of local PCa recurrence after both RP and RT. Mp-MRI joins anatomic and biological information together thanks to the combination of morphological imaging such as T2-weighted imaging (T2WI) and functional techniques such as dynamic contrast-enhanced imaging (DCEI), diffusion-weighted imaging (DWI), and MR spectroscopic imaging (MRSI).

Morphological T2WI is acquired with a high spatial resolution technique (3-4 mm thickness) in order to identify very small pathological tissues [42].

In DCEI the prostate bed is repetitively acquired with a gradient-echo T1W sequence before and after intravenous injection of contrast medium over a period of time. DCEI, in addition to qualitative assessment of the images, allows the calculation of semiquantitative parameters such as peak enhancement, time to peak, wash-out slope, area under the contrast enhancement curve (AUC), and quantitative parameters, such as *K* ^trans⁡^,  *v*_*e*_, and *K*_ep_. PCa shows neoangiogenesis and is, therefore, associated with early and high peak enhancement, wash-out slope, high AUC, and higher *K* ^trans⁡^, *K*_ep_, and *v*_*e*_ than normal peripheral zone [43].

DWI is based on an echo-planar sequence and depicts the diffusivity of water molecules along the three space directions within the tissue. It provides qualitative and quantitative information about “cell density” and cell membrane integrity. In neoplastic prostatic tissue extracellular space is decreased; therefore the movement of water molecules is restricted and the so-called apparent diffusion coefficient (ADC) values are low compared to healthy prostatic tissue. DWI can be performed without the administration of exogenous contrast agent and it does not require long acquisition times, being therefore considered the functional technique more practical, quicker, and simple to use [44]. Diffusion tensor technique (DTI) is another echo-planar imaging technique that exploits the diffusivity of water molecules to map the orientation of submillimetric nerve fibers. Unlike DWI, DTI highlights the diffusivity of water molecules along several space directions within the tissue. To date, DTI tractography has shown promising results in the evaluation of periprostatic nerve plexus in terms of neuroanatomical distribution, density, and relationship with the prostatic capsule. This information could be useful for guiding proper nerve-sparing surgery, thereby ensuring maintenance of erectile function after RP. On the other hand DTI fiber tracking could play an important role in the evaluation of nerve fibers damage after RP or RT [45].

MRSI provides 3-dimensional data set of the prostate gland, with volume voxels ranging from 0.24 cm to 0.34 cm. This functional technique evaluates the relative concentration of metabolites within voxels. The main metabolites in the prostate gland are citrate (Cit, a marker of benign tissue), creatinine (Cr, insignificant for diagnosis, but difficult to resolve from choline), and choline (Cho, involved in the cellular membrane synthesis and degradation, a marker of malignant tissue). Cit is synthesized, stored, and secreted by normal glandular prostatic tissue. Within normal prostate cells, Cit levels are typically higher than other metabolites because the high concentration of intracellular zinc inhibits the citric acid cycle, leading to accumulation of citric acid. Because of the change in this metabolic pathway and loss of ductal morphology, citric acid does not accumulate in PCa cells and Cit levels are drastically reduced in favour of an abnormal increase in Cho [46]. Cit levels are low in well-differentiated PCa and effectively absent in poorly differentiated tumors [47]. The peak integral ratio of Cho plus Cr to Cit (CC/C ratio) can distinguish PCa tissue from healthy glandular tissue. Conforming to the literature, in a nontreated prostate gland, each voxel can be defined as follows: fibrotic or scar tissue when the ratio is <0.2, residual healthy prostatic glandular tissue when the ratio is between 0.2 and 0.5, probably recurrent PCa when the ratio is between 0.5 and 1, and definitely recurrent PCa tissue when the ratio is >1 [48]. Compared with DWI or DCE, MRSI is a more complex functional technique and it also requires longer acquisition times. It is also important to remember that adequate acquisition of spectroscopic data is dependent on the expertise available. Some centers have dedicated spectroscopists who perform both pre- and post-processing of data and manual case-by-case adjustments that result in significantly better spectra than commercially available software. However, most centers do not have the benefit of such dedicated personnel. On the other hand, the acquisition and quality of DWI and DCE tend to be more homogeneous throughout different platforms and institutions.

## 5. Multiparametric-MRI after Radical Prostatectomy

### 5.1. Rationale and Capabilities

With the development of intensity-modulated RT and image-guided RT, there is the potential to escalate the dose in areas of known disease recurrence, so that accurate identification of local recurrence with pelvic imaging might improve the effectiveness of tumour eradication, improving therefore the chance of long-term control [49]. Cross-sectional imaging modalities (ultrasound, computed tomography, and morphologic MRI) have previously been evaluated in the detection of local recurrence following RP, but each of them is poorly sensitive for detecting a small-sized relapse and is unable to distinguish between local recurrence and postsurgical scarring [50–59]. Over the last few years new technological innovations have allowed the development of imaging techniques which link anatomic, functional, and biological information together. Mp-MRI and positron emission tomography/computed tomography (PET/CT) have proven to be emerging techniques useful in the early diagnosis of PCa recurrence.

Currently, in agreement with literature data, the Se and Spe of PET/CT using 11C- or 18F-labeled Cho compounds, in restaging patients with PCa after RP, are greater in detecting metastatic lymph nodes, distant metastases, and local neoplastic recurrences when serum PSA values are >1 ng/mL, PSA_dt_ is <6 months, and PSA_ve_ is >2 ng/mL/year [60, 61]. Although PET/CT is recommended in patients with high PSA serum values, in patients who experience low biochemical alterations after RP (PSA serum values between 0.2 and 1 ng/mL) it is very important to exclude the presence of locoregional recurrence, being this information essential for radiation oncologists. To date, the role of PET/CT in detecting local recurrence in post-prostatectomy bed in patients with BF and low PSA values is still incompletely defined, probably because of the poor detection rate of small lesions, which may be due to the limited spatial resolution (5-6 mm) of PET scanners.

**Figure 1:**
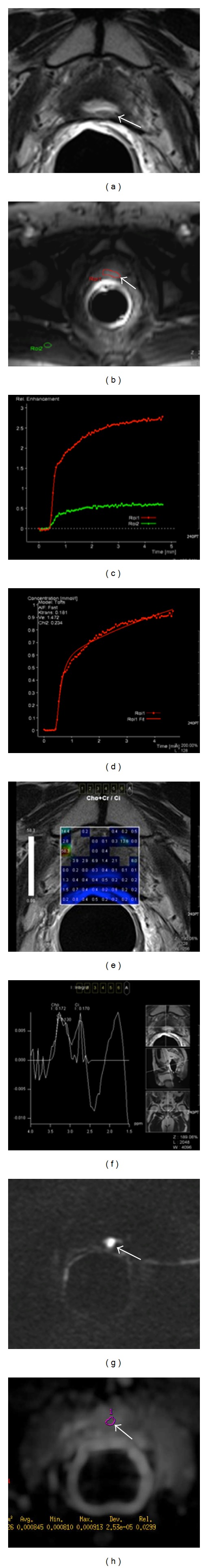
Multiparametric-MR images of a 64-year-old man with prostate-specific antigen progression (PSA serum level 0.75 ng/mL) after radical retropubic prostatectomy, with suspected local recurrence. (a) Axial T2-weighted fast spin-echo image shows a soft tissue nodule of 1 cm in size on posterior perianastomotic location in front of the rectal wall at about 40 mm from the ureteral meatus which is slightly hyperintense compared to pelvic muscles (white arrow). (b) Axial gradient-echo T1-weighted perfusion image showing a remarkable enhancement of the pathological tissue (white arrow). (c) Semiquantitative signal intensity-time curve showing a significant difference between pelvic muscle enhancement (ROI2, green curve) and the higher peak enhancement values of suspected area (ROI1, red curve). (d) Quantitative concentration-time curve of the hypervascular nodule showing a high area under the curve. (e) Color map of choline-creatine to citrate ratio and (f) analysis of spectra show an increased choline-creatine to citrate ratio >0.5. (g) Axial native DWI image at *b* value = 3,000 s/mm^2^ and (h) axial ADC map reconstructed from images obtained at *b* values of 0, 500, 1,000, and 3,000 s/mm^2^ showing marked restricted diffusion (white arrow). All these findings are consistent with locoregional relapse.

**Figure 2:**
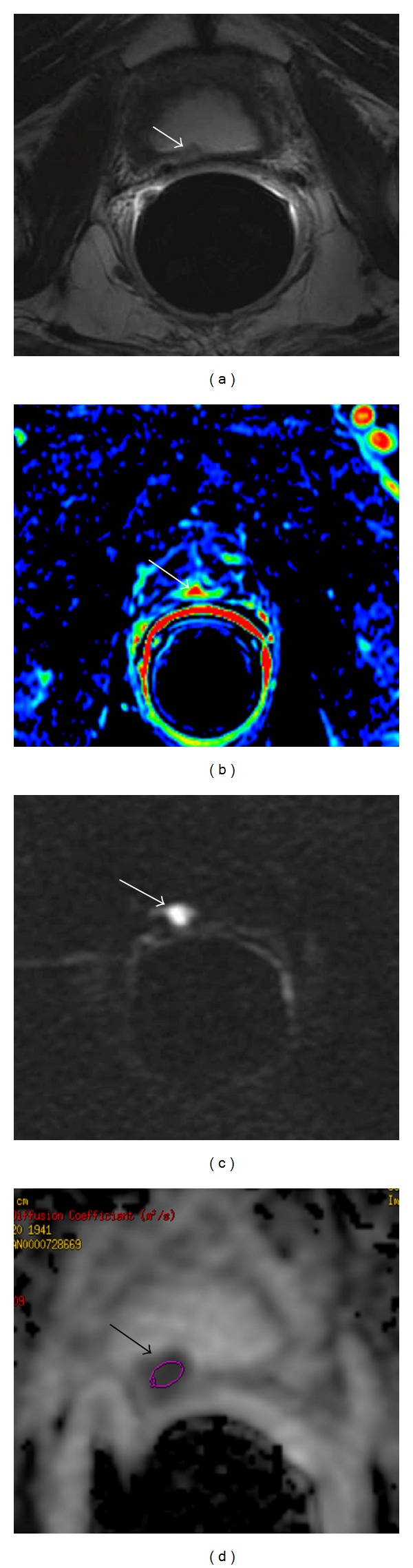
Multiparametric-MR images of a 74-year-old man with prostate-specific antigen progression (PSA serum level 0.43 ng/mL) after radical retropubic prostatectomy, with suspected local recurrence. (a) Axial T2-weighted fast spin-echo image shows a solid nodular tissue of about 7 mm in size on the right posterior perianastomotic location in front of the rectal wall at about 12 mm from the ureteral meatus which is slightly hyperintense compared to pelvic muscles (white arrow). (b) Axial gradient-echo T1-weighted color map image showing a remarkable enhancement of the pathological tissue (white arrow). (c) Axial native DWI image at *b* value = 3,000 s/mm^2^ showing marked restricted diffusion of water molecules (white arrow). (d) Axial ADC map reconstructed from images obtained at *b* values of 0, 500, 1,000, and 3,000 s/mm^2^ shows a dark area corresponding to the abnormal hyperintense tissue seen on T2-weighted images and hypervascular nodule seen on color map (black arrow). All these findings are consistent with locoregional relapse.

MRI—thanks to its inherent superior contrast and spatial resolution, especially with an endorectal coil (ERC)—represents an emerging and promising modality for the evaluation of prostatic fossa after RP. In addition, the recent development of functional MRI techniques has provided promising results for accurate detection and characterization of small recurrent PCa (Figures 1 and 2). Moreover mp-MRI after RP is a very useful tool to discriminate between locoregional relapse and small amount of residual glandular healthy tissue, scar/fibrosis, and granulation tissue and it may even be able to assess the aggressiveness of nodule recurrence by means of ADC values. The presence, on T2W images, of a lobulated, semicircumferential, nodular-like, or plaque-like soft tissue thickening in the prostatectomy bed that appear slightly hyperintense compared to pelvic muscles should be considered to be strongly suggestive of local recurrence [62]. The most common site of postoperative local recurrence is the vesicourethral anastomosis around the urinary bladder and/or membranous urethra [5]. Other common sites of local recurrence are retrovesical (between the urinary bladder and rectum), within retained SVs, at the anterior or lateral surgical margins of the prostatectomy bed (e.g., abutting the levator ani muscles) and at the resection site of the vas deferens [4, 63, 64]. In most cases local recurrence can be readily distinguished from normal perianastomotic scar/fibrotic changes which appear of low signal intensity (SI) compared with muscle on T2W images; however, granulation tissue may occasionally be present in the perianastomotic region, where it can mimic the appearance of tumor recurrence [4]. When conventional T2WI is not able to discriminate between local recurrence and postoperative changes, DCEI is of paramount importance for the differential diagnosis. A recurrent tumor tends to enhance quickly and avidly in the arterial phase, which is followed by a plateau or washout on the SI-curve during the venous phase, while postoperative changes tend to show either no enhancement or mild enhancement in the venous phase [62]. In case of larger lesions in the prostatectomy fossa (>10 mm) MRSI can play a role as problem solving technique in doubtful cases when the other techniques are borderline. A high Cho concentration in the lesion is more suggestive of PCa recurrence than residual benign gland or fibrosis. In a recent paper Panebianco et al., in order to evaluate the aggressiveness of local recurrent PCa, compared ADC values of locoregional recurrences with the histological results [10]. The mean and standard deviation of ADC values were 0.5 ± 0.23 mm^2^/s for high-grade aggressiveness, 0.8 ± 0.09 mm^2^/s for intermediate-grade aggressiveness, and 1.1 ± 1.17 mm^2^/s for low-grade aggressiveness; ADC values higher than 1.3 mm^2^/s (mean ADC values 1.4; range 1.3–1.7) were found in patients with a histological finding of prostatic gland remnants.

**Figure 3:**
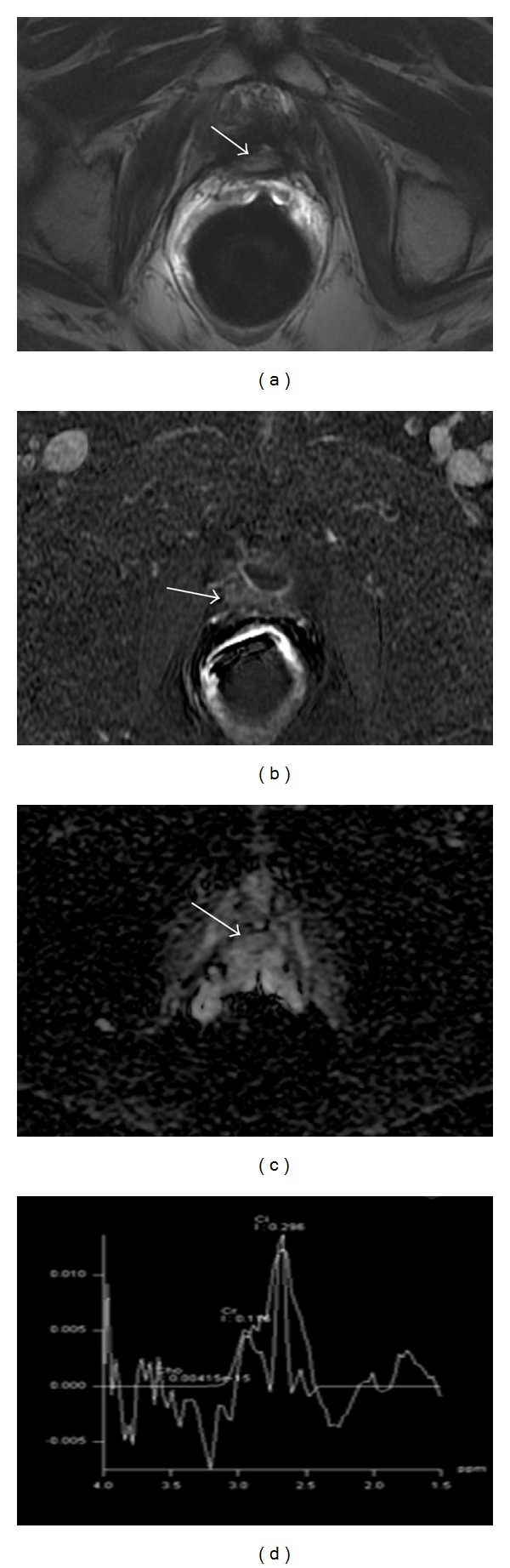
Multiparametric-MR images of a 69-year-old man with prostate-specific antigen progression (PSA serum level 0.6 ng/mL) after radical retropubic prostatectomy, with suspected local recurrence. (a) Axial T2-weighted fast spin-echo image shows a solid nodular tissue of about 8 mm in size on the right posterior perianastomotic location in front of the rectal wall at about 14 mm from the ureteral meatus which is slightly hyperintense compared to pelvic muscles (white arrow). (b) Axial gradient-echo T1-weighted subtracted image shows no signs of enhancement of the abnormal tissue detected on T2-weighted images (white arrow). (c) Axial ADC map reconstructed from images obtained at *b* values of 0, 500, and 1,000 s/mm^2^ shows a bright area corresponding to the abnormal hyperintense tissue seen on T2-weighted images (white arrow). (d) ^1^H-magnetic resonance spectroscopic imaging reveals a choline-plus-creatine-to-citrate ratio lower than 0.3. All these findings are consistent with residual glandular healthy tissue.

In patients scheduled for local salvage EBRT after RP, accurate anatomic localisation of tumour deposits within the post-prostatectomy bed, by means of mp-MRI, may allow for an individualised field of irradiation, thereby maximising efficacy and minimising toxicity to normal surrounding tissues. In this setting mp-MRI findings could be used to apply a stereotactic boost to the recurrence site, potentially improving in this way the control of local disease and avoiding further locoregional relapses over time. Furthermore, the differential diagnosis between residual glandular healthy tissue and locoregional neoplastic recurrence is of paramount importance for the radiation oncologist because the dose of RT delivered in the prostate bed is quite different (Figure 3) [65, 66].

The recent development of the new hybrid PET/MRI scanners, with simultaneous acquisition of mp-MRI and PET images, can yield combined structural, functional, and metabolic information that can potentially affect patient management and outcome [67]. Cho-PET/MRI might improve RT planning by enabling more precise target volume delineation of local recurrence as well as of PCa involved lymph nodes [68].

### 5.2. Multiparametric-MRI Evidence after Radical Prostatectomy

The characteristics of the different reviewed studies on mp-MRI for the diagnosis of local recurrence after RP are summarized in Table 1.

In 1997 Silverman and Krebs showed that MRI had a sensitivity (Se), a specificity (Spe), a positive predictive value (PPV), and a negative predictive value (NPV) of 100% for detecting local PCa recurrence in patients with clinical suspicion of locally recurrent disease after RP [3]. They enrolled 41 men, 35 of which had clinical suspicion of PCa local recurrence either from a new palpable nodule or induration in the prostatic bed or an elevated PSA serum level (range, 0.4 to 11 ng/mL). They used an imaging protocol composed by T2WI as well as axial T1W unenhanced and gadolinium-enhanced MR images. TRUS-guided biopsy of the prostate bed was used to validate MRI results. Thirty-one of the 35 men with clinical suspicion of PCa local recurrence had a distinct enhancing soft-tissue nodule (range, 7–38 mm) in the prostatic bed; all 31 of these soft-tissue nodules were histologically proven PCa local recurrence. No distinct abnormal tissue was seen on MR images of the remaining 4 patients with clinical suspicion of PCa local recurrence and biopsy of the prostatic bed revealed fibrosis. The 6 patients with no clinical evidence of locally recurrent PCa had no distinct nodule in the prostatic bed on MR images. A major weakness of these too promising results is that this report lacks a real true-negative group of patients because negative TRUS-guided biopsy results do not imply that no locally recurrent PCa is present as a false-negative result may be caused by sampling error. In later reports, however, significantly lower accuracies were found, probably due to larger study populations and the inclusion of smaller and less clinically evident local recurrences (at lower PSA values), resulting in lower Ses and lower Spes.

Sella et al. reviewed the unenhanced MR images of 48 patients with BF after RP (PSA ranging from undetectable to 10 ng/mL) [4]. Local recurrence was considered present if there was no evidence of distant metastases and there was a positive biopsy result, subsequent reduction in serum PSA values after RT of the pelvis, or serial MR findings that demonstrated at least a 20% increase in the size of a suspicious pelvic soft tissue mass. In 39 patients, MRI demonstrated at least one soft tissue mass in the post prostatectomy fossa (maximum transverse diameter ranging from 0.8 to 4.5 cm). The Se and Spe of unenhanced MRI in detecting loco-regional relapse were, respectively, 95% and 100%. The major concern of these results is that they were reached with a small patient cohort with a very large size of local recurrence and very high serum PSA levels.

Further studies confirmed the value of mp-MRI in depicting local recurrent PCa in patients with BF after RP. Cirillo et al. showed that DCE improves the diagnostic performance in detecting local PCa recurrence in comparison to unenhanced imaging [5]. They enrolled 72 patients (mean total serum PSA range: 0.2–8.8 ng/mL). The standard of reference for local recurrence was as follows: positive biopsy findings in the prostatectomy bed, ^11^C-Cho PET positive in the prostatectomy bed, and reduction of the PSA values after pelvic radiotherapy; a patient was considered to be positive when at least one of these criteria was met. The diameter of local recurrences detected on the unenhanced images varied from 0.8 to 4.2 cm. The size of the local relapses detected on DCEI ranged from 0.8 to 3.5 cm. Se, Spe, PPV, NPV, and accuracy in detecting locoregional relapse were, respectively, 61.4%, 82.1%, 84.4%, 57.5%, and 69.4% for unenhanced MRI and 84.1%, 89.3%, 92.5%, 78.1%, and 86.1% for DCEI. The discrepancy in the values of Se and Spe from previous studies could be explained by the larger sample size and the lower rate of clinically evident local recurrences (i.e., 84.1% versus 95% in Sella's study versus 100% in Silverman's study).

Casciani et al. confirmed that DCEI improves diagnostic performance in comparison to unenhanced MRI [6]. They enrolled 46 patients with PSA ranging from 0.1 to 6 ng/mL. The average maximum diameter of soft-tissue nodules detected on MRI was 1.5 cm (range, 0.4 to 4.0 cm). TRUS-guided biopsy or reduction in serum PSA level after RT was used to validate MR results. Overall, unenhanced MRI showed Se 48%, Spe 52%, PPV 54%, NPV 46%, and a diagnostic accuracy of 48%. DCEI displayed Se 88%, Spe 100%, PPV 100%, NPV 88%, and a diagnostic accuracy of 94%.

Although the abovementioned studies were based on a considerable number of patients and the mean serum PSA level was not very high, their accuracy is partially limited by the average size of local PCa recurrence which is always greater than 1.5 cm. Moreover these studies do not evaluate ^1^H-MRSI or DWI technique.

Sciarra et al. [7] showed that combined ^1^H-MRSI and DCEI allow higher diagnostic accuracy in identifying local PCa recurrence. They enrolled 70 consecutive male patients. The population was divided into two groups: group A (50 patients) where the presence of local disease was ascertained on the basis of TRUS-guided biopsy and group B (20 men) where a reduction in PSA level >50% following RT was used to validate MR results. In group A PSA serum value ranged from 0.9 to 1.9 ng/mL and the size of the suspicious local recurrence ranged from 7.6 to 19.4 mm. In group B serum PSA level ranged from 0.4 to 1.4 ng/mL and the maximal transverse dimension of a suspect local recurrence varied from 5.0 to 7.2 mm. In group A ^1^H-MRSI alone showed Se 84%, Spe 88%, PPV 93%, and NPV 74%; DCEI alone had Se 71%, Spe 94%, PPV 96%, and NPV 73%; combined ^1^H-MRSI-DCEI displayed Se 87%, Spe 94%, PPV 96%, and NPV 79%. Areas under the receiver operating characteristic curve (*A*_*z*_) for ^1^H-MRSI, DCEI, and combined ^1^H-MRSI-DCE were 0.942, 0.931, and 0.964, respectively. In group B, ^1^H-MRSI alone showed Se 71%, Spe 83%, PPV 91%, and NPV 56%; DCEI displayed Se 79%, Spe 100%, PPV 100%, and NPV 67%; combined ^1^H-MRSI and DCEI had Se 86%, Spe 100%, PPV 100%, and NPV 75%. *A*_*z*_s for ^1^H-MRSI, DCEI, and combined ^1^H-MRSI-DCEI were 0.810, 0.923, and 0.940, respectively.

In a recent study Panebianco et al. found that ^1^H-MRSI-DCEI combined technique showed higher Se, Spe, and accuracy than ^18^F-Cho PET/CT in the identification of small lesions in patients with low BP after RP (serum PSA values ranging from 0.2 to 2 ng/mL) [8]. Eighty-four consecutive male patients were enrolled in the study. Patients were divided into two groups on the basis of the clinical validation used for MR and PET/CT results. The “gold standard” for local disease was a reduction in serum PSA level >50% following RT in group A and TRUS-guided biopsy in group B. Group A included 28 patients with PSA ranging 0.8–1.4 ng/mL and maximal transverse dimension of the lesions ranging from 5 to 7.2 mm. Group B included 56 men with PSA serum level ranging from 1.3 to 2.5 ng/mL and lesion size ranging from 7.6 to 19.4 mm. In group A combined ^1^H-MRSI-DCEI showed Se 92%, Spe 75%, PPV 96%, NPV 60%, and accuracy of 89% in identifying local recurrence, while PET-CT displayed Se 62%, Spe 50%, PPV 88%, NPV 18%, and accuracy of 60%. The *A*_*z*_s for MR and PET-CT values was 0.833 and 0.562, respectively. In group B combined ^1^H-MRSI-DCEI showed Se 94%, Spe 100%, PPV 100%, NPV 57%, and accuracy of 94%, whereas PET-CT had Se 92%, Spe 33%, PPV 98%, NPV 43%, and accuracy of 91%. The *A*_*z*_s for MR and PET/CT values was 0.971 and 0.837, respectively. These last two recent studies were based on a notable number of patients and detected tumor recurrences less than 1.5 cm in size.

Wu et al. in a meta-analysis of the aforementioned studies carried out to assess the effectiveness of mp-MRI in detecting local recurrent PCa after RP found that DCEI, compared to T2WI, showed higher pooled Se (85%) and Spe (95%) and when combined with MRSI had the highest pooled Se (92%) [9].

On the basis of these studies, DCEI can be considered as the most reliable MRI technique for the detection of local PCa recurrence after RP. However it must be taken into account that vascularity and contrast enhancement can be reduced in patients who have received ADT, which may limit the application of this technique. The addition of MRSI to DCE can significantly improve the diagnostic accuracy of local PCa recurrence detection. Despite these promising results, the role for spectroscopy after RP remains controversial and needs to be defined further. Indeed, spectroscopy is limited by its poor spatial resolution and its high sensitivity to field inhomogeneities and susceptibility artifacts caused by surgical clips in the anastomotic area, which decrease the spectroscopic quality and can preclude successful spectroscopic measurements. Moreover, the best diagnostic criteria are still unclear as normal Cit is in theory undetectable after RP and thus the classic CC/C ratio might not be accurate [9]. Furthermore metabolite signal-to-noise ratio (SNR) depends on factors that could differ among voxels in the same patient or among patients (e.g., the distance of the voxels from the ERC, the magnetic field homogeneity, and the T2 relaxation times); therefore it should be recognized that these factors could introduce bias against ^1^H-MRSI data for the differentiation between benign/fibrotic tissue and persistent/recurrent PCa [7].

All the above-mentioned studies have a big weakness because none of them compares DCEI with DWI. In a post-prostatectomy setting the low SI of the bladder and the prostatic bed on high-*b*-value images ensures that only recurrent PCa tissue appears bright. DWI requires no special software for image analysis and no particular experience in image interpretation, since visualization of local recurrence is straightforward. Nevertheless a current limitation of this technique is the lack of standardization [69, 70]. In a recent study Giannarini et al. described 5 patients with BF after RP and pelvic lymph node dissection in whom conventional MRI findings were negative or equivocal and locoregional relapse was depicted with DWI; all TRUS-guided biopsy cores directed to the lesions discovered on DW images were positive for malignant prostatic tissue [70].

Panebianco et al. [10] in order to validate the role of 3-T DWI in the detection of local PCa recurrence analyzed a large number of patients (262 men) with BP after RP. The patient population was divided into two groups according to recurrent lesion size detected on MRI and PSA serum level. Group A included 126 patients with PSA ranging from 0.5 to 1.7 ng/mL and a lesion size ranging from 4 to 8 mm. Group B included 116 patients with PSA serum level ranging from 1.4 to 2.9 ng/mL and a lesion size ranging from 9 to 15 mm. In group A the presence of local disease was ascertained on the basis of TRUS-guided biopsy, while in group B a reduction of PSA serum values higher than 50% following RT was used to validate MR results. For the identification of local recurrence in group A, combined T2WI and DCEI (T2 + DCE) displayed Se 98%, Spe 94%, PPV 97%, NPV96%, and accuracy of 93%; combined T2WI and DWI with a *b* value of 3,000 mm^2^/s (T2 + DW3) had Se 97%, Spe 95%, PPV 96%, NPV 95%, and accuracy of 92%; combined T2WI and DWI with a *b* value of 1,000 mm^2^/s (T2 + DW1) showed Se 93%, Spe 89%, PPV 94%, NPV 91%, and accuracy of 88%. The *A*_*z*_ for T2 + DCE was 0.917, for T2 + DW3 was 0.823, and for T2 + DW1 was 0.724. In group B T2 + DCE had Se 100%, Spe 97%, PPV 96%, NPV 95%, and accuracy of 91%; T2 + DW3 showed Se 98%, Spe 96%, PPV 93%, NPV 91%, and accuracy of 89%; T2 + DW1 displayed Se 94%, Spe 92%, PPV 91%, NPV 89%, and accuracy of 86%. The *A*_*z*_ for T2 + DCE was 0.875, for T2 + DW3 was 0.783, and for T2 + DW1 was 0.679. The authors supposed that the overall accuracy of DCEI is superior to that of DWI because DW images are more affected by distortion artifacts due to surgical clips and background noise than DCE images are, though there are some cases in which DCEI is doubtful and DWI is of paramount importance for local recurrence depiction. For instance, a prominent periprostatic venous plexus may sometimes mimic the appearance of enhancing recurrent tumor on DCE images; therefore when there is this potential pitfall DWI is mandatory to exclude the presence of abnormal tissue in the post-prostatectomy bed.

The limitation of all the aforementioned studies is that none of them presented an analysis of all functional techniques available on a mp-MRI examination of the prostatic bed. Roy et al. in a recent study evaluated the Se of the three types of functional MRI techniques in the detection of local PCa recurrence after RP and after EBRT [11]. They enrolled 60 consecutive male patients with BF after RP or after EBRT. TRUS-guided biopsy was used to validate MRI results. The patient population was divided into two groups according to the therapy delivered. Group A included 28 patients (serum PSA value range: 0.3–2.8 ng/mL) who underwent RP, and group B included 32 patients (PSA serum level range: 2.2–4.8 ng/mL) who received EBRT. In group A the Se was highest for T2WI plus DCE (97%) followed, in decreasing order, by DCEI alone (94%) and T2WI plus DWI plus DCEI (94%), T2WI plus DWI plus DCEI plus MRSI (74%), DWI alone (65%) and T2WI plus DWI (65%), T2WI alone (56%), T2WI plus MRSI (53%), and lastly MRSI (50%). The worst results were obtained with isolated T2WI and MRSI; the lower performance of MRSI may reflect a partial volume effect due to the voxel size. Although Sciarra et al. reported a Se of 71–84% and a Spe of 83–88% in the detection of local recurrence after RP using a similar MRSI sequence, the results of Roy's study are disappointing most likely because Cit could not be measured since after removal of prostatic gland, a metabolite ratio is difficult to calculate.

## 6. Multiparametric-MRI after Radiation Therapy

### 6.1. Rationale and Capabilities

In patients with local recurrence after RT, if local salvage therapy is not undertaken early, then the median time to development of distant metastases is ≈3 years [71]. Local salvage therapies with curative intent include additional irradiation of the prostate, RP, and other new treatment options such as cryosurgery, transrectal high-intensity focused US, photodynamic therapy and radiofrequency interstitial, and microwave thermoablation [72]. Because the exact location of the recurrent tumor within the prostate is generally unknown, the general practice of salvage therapies involves treatment of the entire prostate [73]. Currently there is an increasing need of imaging techniques able to identify and localize recurrent PCa in order to perform focal salvage therapies effectively with minimal complications. For instance, if salvage RT is scheduled, the detection and accurate localization of recurrent cancer give the potential to reduce the target volume to the recurrent tumor only with a consequent reduction in the treatment-related toxicity and morbidity compared to conventional whole prostate gland salvage radiotherapy [73].

Several studies have reported that TRUS is unreliable for the detection of cancer recurrence after EBRT, showing Se 49% and Spe 57%, which is not superior to digital rectal examination (Se 73%, Spe 66%) [74, 75]. TRUS-guided sextant biopsy, commonly proposed as the reference standard for detection of local recurrence, may require repeated biopsies to reach a final diagnosis [32, 76]. In addition to false-negative results due to sampling error, false-positive results may also occur, because the presence of malignant cells in biopsy specimens may represent biologically inactive tumour remnants, especially in the first 1-2 years after RT [76].

Cho and acetate labeled PET/CT has shown promise in the identification of regional and distant metastases but cannot allow precise location of the intraprostatic post-RT recurrent cancer due to its poor spatial resolution [77].

**Figure 4:**
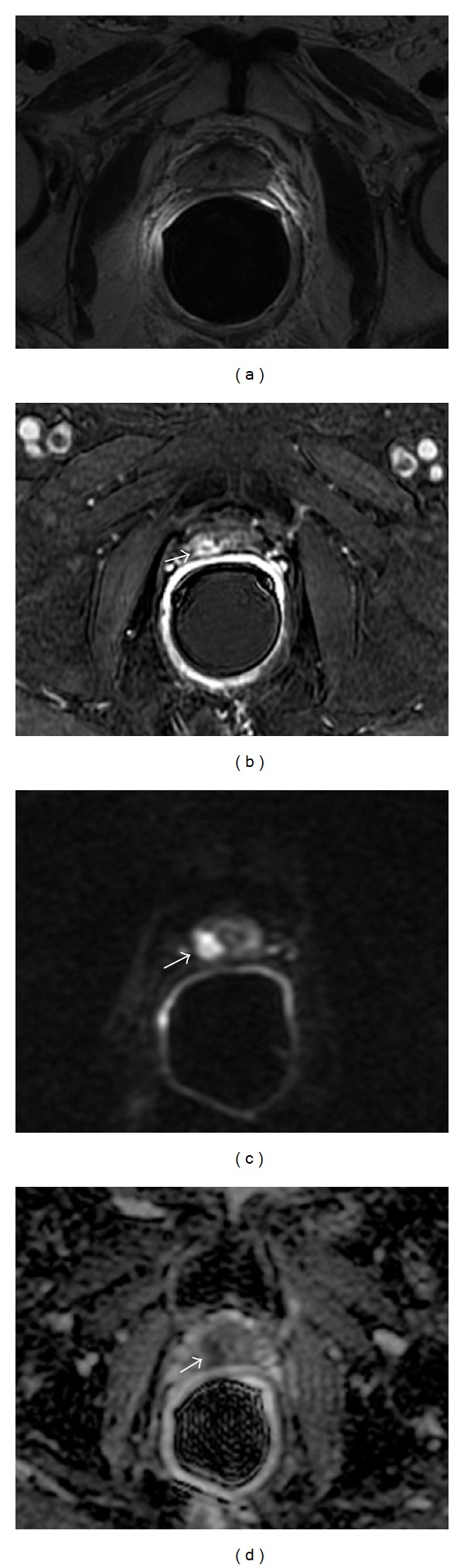
Multiparametric-MR images obtained in a 69-year-old patient with TRUS-guided biopsy-proved PCa (Gleason score, 3 + 4) in the right apex-midgland after 14 months after external beam radiation therapy. (a) Axial T2-weighted fast spin-echo image shows shrinkage of the prostate gland which appears diffusely hypointense because of radiation induced atrophy and fibrosis. No suspicious foci are seen. (b) Axial gradient-echo T1-weighted subtracted image showing a remarkable nodular enhancement at the right apex-midgland (white arrow). (c) Axial native DWI image at *b* value = 3,000 s/mm^2^ showing a focus with marked restricted diffusion (white arrow). (d) Axial ADC map reconstructed from images obtained at *b* values of 0, 500, 1,000, and 3,000 s/mm^2^ shows a dark area corresponding to the hypervascular nodule (white arrow). All these findings are consistent with local recurrent PCa.

At present MRI is widely considered to be the state of the art in detecting and localizing PCa recurrence in patients with BP after definitive RT (Figure 4). After RT, the entire prostate and the SVs show decreased size and diffusely decreased SI on T2WI, and the peripheral, central, and transition zones appear less distinct from each other [78]. PCas also show changes, which may include decreased size, reduced capsular bulging, capsular irregularity, or decreased extracapsular extension. These changes are caused by RT induced glandular atrophy and fibrosis. The effects of RT on the T2WI appearance of adjacent anatomic structures include increased bladder and/or rectal wall thickness, thickening of the perirectal fascia, and increased SI of the pelvic sidewall musculature [79]. In addition, there may be increased SI in the bone marrow on T1W images due to post-RT fatty replacement [79]. T2WI alone is of a limited diagnostic accuracy because the recurrent tumor and the normal surrounding parenchyma both appear hypointense [13]. It has been hypothesized that cancer can be detected under such circumstances if it produces an additional focal reduction in SI [75] or if appears as a hyperintense region compared to surrounding prostate tissue [80]. Moreover, a focal T2 hypointense region may represent the treated nonviable tumor and not necessarily cancer recurrence [19]. Sala and colleagues' data [12] did not support the observation that T2WI is of a limited use in detecting local recurrence after RT. In their study using whole-mount section radiologic-pathologic correlation, they found that the accuracies of T2WI as estimated with *A*_*z*_, Se, and Spe were similar to those obtained in studies [81–83] of untreated patients, in which Ses ranged from 67% to 88%. Westphalen et al. [13], resting upon the observation of recovery of the usual zonal anatomy and normal metabolism at MRSI after RT and/or ADT, investigated if the accuracy of T2WI was influenced by the time interval between RT and MRI. In their study two radiologists analyzed independently 25 patients within 3 years of EBRT and 34 more in the 3 years after therapy, using TRUS-guided biopsy as standard of reference. Logistic regression failed to demonstrate a statistically significant difference in the ability of T2WI to detect cancer based on whether patients were imaged before or after 3 years (reader 1, *P* = 0.86; reader 2, *P* = 0.44), thus concluding that T2WI has low accuracy in the detection of PCa after EBRT, irrespective of the time since therapy.

DCEI, DWI, and MRSI increase the accuracy of MRI in detecting post-RT local recurrent PCa.

### 6.2. Multiparametric-MRI Evidence after Radiation Therapy

A considerable number of studies have explored the potential of functional techniques to improve the MRI assessment of local recurrent PCa after definitive RT (Table 2). Although the results have been promising, the main limitation of these studies is the imperfect standard of reference used. Indeed most authors used core biopsy results as the standard of reference rather than histologic step-section analysis of salvage prostatectomy specimens. The limitations of biopsy in PCa detection for both newly diagnosed and treated PCa have been extensively discussed and documented in the literature [76, 84]. Indeed TRUS-guided biopsy, even with an extended or saturation method, is subject to sampling error. Therefore although mp-MRI results are very promising for planning local salvage therapies targeted to positive regions, further whole-mount radiologic-pathologic correlation studies would be required to definitively validate the role of mp-MRI in detecting and localizing local PCa recurrence. However, it is quite challenging to perform this correlation because salvage RP is seldom performed, given the high rate of complications and the patient population, which tends to be composed by elderly with multiple comorbidities.

The first studies compared DCEI and T2WI. Identifying a recurrent disease using DCEI is easier, paradoxically, than the initial detection of cancer: this is due to the very different patterns between recurrence and postradiation fibrosis [35]. After RT, recurrent tissue can be recognized as hypervascular early enhancing homogeneous nodule, whereas in the surrounding prostatic tissue the enhancement is homogeneous, slow, and low [85]. Hence, DCEI is more reliable than T2WI for the detection of recurrent PCa. However, this functional technique is not standardized yet. Various temporal resolutions have been used (5–95 s) and the best compromise between spatial and temporal resolution remains to be found. A serious drawback of DCEI is that its Spe in depicting the recurrence in the central gland is almost always reduced. Areas showing increased perfusion in the prostate central gland, representing angiogenesis in benign prostatic hypertrophy, can result indeed in a confusing visualization of recurrent tumours [73]. DCEI is also a useful tool to monitor response to therapy because PCa may undergo a decrease in tumor vascular permeability that can be assessed both with a qualitative and quantitative approach [86, 87]. DCEI should be performed at least 3 months after RT because an increase in perfusion and blood volume due to an inflammatory reaction of the tissue to radiotherapy can be found immediately after treatment.

Rouvière and colleagues [14] compared conventional MRI and DCEI in 22 patients, using biopsy results as the reference standard (PSA level range: 1.01 to 21 ng/mL). They achieved higher Ses with DCEI than they achieved with conventional T2WI (0.70–0.74 versus 0.26–0.44), while the Spes they achieved with the two techniques were similar (0.73–0.85 versus 0.64–0.86). Nevertheless, while the differences between DCEI and T2WI with respect to Se were statistically significant, the differences with respect to Spe were not. In addition the interobserver agreement was greater for DCEI (*k* = 0.63–0.70) than for the T2WI (*k* = 0.18–0.39).

Haider et al. [15] confirmed that DCEI performs better than T2WI in the detection and localization of PCa in the peripheral zone after EBRT. They enrolled 33 patients with BF after EBRT (PSA range: 0.1–11.7 ng/mL). Systematic TRUS-guided biopsy was used as the reference standard for the presence or absence of tumor. DCEI had significantly better Se (72% versus 38%), Spe (85% versus 80%), PPV (46% versus 24%), NPV (95% versus 88%), and accuracy (83% versus 74%) than T2WI.

Kara et al. [16] examined 20 patients with biopsy proven local recurrence after EBRT and found that the accuracy of the DCEI in the detection of recurrence was significantly higher compared to that obtained using T2WI. In the detection of tumor recurrence TRUS imaging had Se 53.3%, Spe 60%, PPV 80%, NPV 30%, and accuracy of 55%; T2WI displayed Se 86%, Spe 100%, PPV 100%, NPV 71%, and accuracy of 90%; DCEI showed Se 93%, Spe 100%, PPV 100%, NPV 83.3%, and accuracy of 95%.

It is of note that, in the aforementioned studies, very simple visual criteria were used for DCEI with quite low temporal resolutions (30–96 s). This suggests that the detection of local recurrences using DCEI is easy and can be done on standard MR scanners without dedicated software.

Yakar et al. [80] evaluated the feasibility of MR-guided biopsy in the diagnosis of recurrent PCa after EBRT. They enrolled 24 patients with BR. T2WI and DCEI were used to localize tumour suspicious regions (TSRs) for biopsy. Of the 38 different TSRs identified on MR images, 26 contained histologically proven recurrence (68%), 8 revealed radiotherapy induced atypia (21%), 1 contained residual indeterminate PCa with severe radiation changes (3%), and the remaining 3 contained fibrosis (8%). These results showed that local recurrence after EBRT could be localized with the combination of MR-guided biopsy and diagnostic MRI in a substantial proportion of patients (PPV 68% and 75% on a per TSR and a per patient basis, resp.). The authors concluded that with a median intervention time of 31 minutes, and no procedure-related complications, MR-guided biopsy can be considered a feasible method in localizing and diagnosing local PCa recurrence using a low number of cores compared to TRUS-guided biopsy in a clinically acceptable time (3 biopsy cores versus 6–22 cores).

Other studies investigated the role of MRSI in evaluating the response to ADT or RT and in detecting local recurrence and yielded promising results. Voxels with spectra containing no significant metabolite peaks, specifically spectra having peak area-to-noise ratio <5 : 1 for Cho, polyamines, Cr, and Cit, are considered free of metabolites and represent the so-called metabolic atrophy which is highly indicative of a successful and effective treatment. The use of an ERC is essential for spectroscopic imaging even at high field strength, because it increases SNR by a factor of 10, which results in higher spatial and spectral resolution [88]. It is known that Cit and polyamine spectral peaks decrease rapidly and progressively over time after EBRT or ADT, at a faster rate than Cho and Cr levels [89], because Cit is a biomarker of healthy cells which go first into apoptosis than cancerous cells. Thus, measuring the levels of Cit in these patients is of limited use for the identification of cancer recurrence. Moreover it has been shown that in biopsy specimens obtained after EBRT and studied with high-resolution MRSI, Cit was undetectable in almost all benign and malignant biopsy specimens [17]. The reparative proliferation of prostatic parenchyma after radiation-induced cellular damage seems to promote the conversion of the energy metabolism in the prostate from Cit-producing to Cit-oxidizing, resulting in suppressed Cit metabolism and secretion, and to increase the demand for Cho involved in the phospholipid cell membrane synthesis and degradation. As a result, Cit tends to decrease and Cho tends to increase, either in benign as in malignant areas, thus causing false positive findings [19]. Therefore, the CC/C ratio, which is used in the characterization of cancer in nontreated patients, could be of limited use after RT. In this context, the normal metabolic background is lacking, and it remains unclear what metabolic criteria should be used to differentiate benign from malignant areas in an irradiated prostate gland. Some authors define persistent or recurrent disease as Cho-to-Cr ratio >1.5 : 1 if Cr is detectable, and Cho peak area-to-noise ratio >5 : 1 if Cr is undetectable, whereas others use the standard CC/C ratio to discriminate between benign prostatic tissue from persistent/recurrent disease. For instance Panebianco et al. [90] in a recent study showed that MRSI followup, using CC/C ratio, shows a greater potential compared to PSA in monitoring patients after EBRT because MRSI can demonstrate PCa recurrence or residual disease before the BR occurs, leading to the possibility to deliver salvage local therapy, and thus the chance for cure as early as possible.

Menard et al. [17], in an ex vivo study, showed that, although the spectral features of prostate tissue markedly change after radiotherapy, MRSI can accurately identify histologically malignant biopsies. They enrolled 35 patients who underwent EBRT and a TRUS-guided biopsy. One hundred sixteen tissue specimens were subjected to ^1^H-MRSI and histopathologic analysis. The Se and Spe of MRSI in identifying a malignant biopsy were 88.9% and 92%, respectively, with an overall classification accuracy of 91.4%.

Coakley et al. [18] demonstrated that MRSI is substantially more accurate than conventional T2WI in the detection of post-RT local recurrence. They enrolled 21 patients with BF (serum PSA level range: 0.4–4.8 ng/mL). Sextant biopsy was used as the standard of reference. The *A*_*z*_ for the detection of locally recurrent cancer with T2WI was 0.49 for reader 1 and 0.51 for reader 2, indicating only slight interobserver agreement (weighted *K* = 0.20). The *A*_*z*_ for the detection of locally recurrent cancer with MRSI was 0.81, significantly greater than the *A*_*z*_ at T2WI (*P* < 005). The finding of 3 or more suspicious voxels in a hemiprostate had a Se and Spe of 89% and 82%, respectively, for the diagnosis of locally recurrent PCa. A weakness of this study is that MR spectral analysis was confined to peripheral-zone voxels only, thus potentially missing transitional zone recurrences.

Pucar et al. [19] assessed the role of T2WI, MRSI, digital rectal examination, and sextant biopsy in detecting local recurrence after EBRT, using whole-mount section pathologic findings from salvage RP as the standard of reference. They enrolled 9 patients with increasing PSA levels after EBRT who underwent a salvage RP. On the basis of published data in untreated and hormone-treated gland [91] voxels were considered suspicious tumor in the peripheral zone if they had a CC/C ratio of 0.5 or more. T2WI and MRSI showed Se of 68% and 77%, respectively, in detecting local recurrence, while Se of biopsy and digital rectal examination were 45% and 16%, respectively. MRSI appeared to be less specific (78%) than the other 3 tests, each of which had a Spe higher than 90% (T2WI 96%; sextant biopsy 95%; DRE 96%). MRSI had higher Se (77%) but lower Spe (78%) than other diagnostic tests. MRSI analysis showed that metabolically altered benign gland could be identified falsely as cancer using the criteria adopted in this study. This could be the reason for the apparently lower Spe of MRSI in comparison to the other diagnostic texts. Anyway, despite the low Spe of MRSI and the small sample size, on the basis of these data, concordant suspicious T2W images and MRSI findings (a nodular region of reduced SI at T2WI displaying abnormal metabolism) strongly suggest local PCa recurrence.

Coakley's and Pucar's studies were based on a small patient cohort and did not assess the diagnostic performance of combined T2WI and MRSI in detecting local recurrence after definitive EBRT. Westphalen [20] and colleagues confirmed that the addition of MRSI to T2WI significantly improves the diagnostic accuracy. They retrospectively analysed 64 men who underwent MRI exam and TRUS-guided biopsy for suspected local PCa recurrence after definitive EBRT (PSA level range: 0.7–23.3 ng/mL). They found a significant difference (*P* = 0.001) between *A*_*z*_ values for T2WI alone (0.67) and those for the integrated approach involving both T2WI and MRSI (0.79).

Wu et al. in a meta-analysis carried out to assess the effectiveness of T2WI, DCEI, and MRSI in detecting local recurrent PCa after EBRT found that DCEI, compared with T2WI, showed higher pooled Se (90%) and Spe (81%). DCEI combined with MRSI had the highest pooled Se and Spe (90%) [9]. Furthermore, a comparison of pre-EBRT and post-EBRT MRI has shown that most recurrent cancers occurred at the site of primary tumours [92].

Other studies evaluated the usefulness of DWI post-RT. After radiotherapy, changes in ADC values occur, this parameter being inversely correlated with changes in cellularity. An increase in ADC values reflects increased water mobility through cell lysis and consequent loss of membrane integrity or an increase in the proportion of total extracellular fluid due to a decrease in cell size or number, whereas a decrease in ADC values reflects decreased free extracellular water movement due to an increase of total cellular size or number [93]. The significant difference in ADC values between the tumors and benign tissues before radiotherapy disappears after treatment. One possible explanation is that, after radiotherapy, benign tissues might show histological changes such as acinar distortion, atrophy, stromal fibrosis with granulation tissue formation, and inflammatory swelling of prostate cells, which might result in a decrease in ADC values, whereas the tumour shows an increase of ADC values. The SNR of DWI at 1.5-T is low not only because of T2 shortening in tissues after radiation fibrosis but also because phased-array coil examinations (common in routine practice) due to their inherently low signal return, have insufficient SNR to robustly quantify ADC from DWI sequences. The use of an ERC improves SNR and allows ADC quantification [23]. The use of 3-T could have in this setting several advantages compared with the standard magnetic field of 1.5-T. Theoretically, the SNR increased twofold on moving from 1.5 to 3 T, and an increased SNR can be translated into improvements in spatial, temporal, and spectral resolution. A limited SNR at 1.5-T may impair MR Se for subtle changes in ADC values of the prostate. The increase in SNR from 3-T imaging enables consequently an increase in the SNR of the ADC maps, so a possible increase in the accuracy of MRI for PCa localization and of the measurements of ADC values may be expected. Therefore, the potential measurement error for tumor ADC values at 3-T might be lower than that at 1.5-T. Several clinical studies have reported DWI as a useful noninvasive technique which provides qualitative (by visual assessment) and quantitative information (by means of ADC values) for measuring therapeutic response in patients with PCa during and after radiotherapy [94, 95]. Song et al. [96] showed that DWI at 3-T could be considered as a feasible and reproducible imaging biomarker to evaluate the early therapeutic changes of PCa, even 1 week after initiating radiotherapy. Indeed their results demonstrate that after an effective RT, the mean ADC values of PCa are increased significantly relative to pretherapy, and DWI revealed early ADC changes in the tumors 1 week after initiating radiotherapy. On the other hand, because a decrease in ADC values after RT can occur both in local recurrence and in stromal fibrosis, DWI could be regarded as an unreliable technique in detecting residual tumour or recurrence. Despite this assumption, the degree of diffusion restriction after radiotherapy in prostate tissue was not as great as expected from other tissue types [97], making this technique useful for detecting locally recurrent PCa in clinical practice. Local PCa recurrence appears as an area of high SI on DWI native images and of low SI on ADC maps relative to the surrounding healthy prostate tissue. Many false positive can occur. Similar findings may be observed in various benign conditions of the prostate, including hemorrhage, hyperplasia, adenoma, and chronic inflammation. Post-biopsy hemorrhage has been also reported to reduce ADC values [98]. At present there is no consensus regarding the optimal *b* value for the detection of PCa. Higher *b* values offer better contrast between malignant neoplasms and benign tissues [99] and can increase the Se of diffusion by diminishing the hyperintensity of the tissues with long T2 relaxation times (i.e., T2 shine-through); however, high *b* values lower the SNR [100] and can decrease the absolute differences in SI between cancerous and normal tissues.

Tamada et al. [21] showed that combined T2WI, DWI, and DCEI provide a sensitive method to detect local recurrence after high-dose-rate (HDR) brachytherapy (BT). They included in the study 16 men with BF (PSA range: 2.93–26.59 ng/mL). 12-core-specimen TRUS-guided biopsy was used as the standard of reference. For predicting recurrent cancer, T2WI showed Se 27%, Spe 99%, PPV 86%, NPV 87%, and accuracy of 87%; DWI had Se 68%, Spe 95%, PPV 75%, NPV 94%, and accuracy of 91%; DCEI displayed Se 50%, Spe 98%, PPV 85%, NPV 90%, and accuracy of 90%, whereas the combination of all the 3 techniques showed Se 77%, Spe 92%, PPV 68%, NPV 95%, and accuracy of 90%.

Kim et al. [22] in a preliminary experience found that for predicting locally recurrent PCa after RT, the use of combined T2WI and DWI showed a better diagnostic performance compared to T2WI alone. They analyzed 36 consecutive patients with an increased PSA level after RT (PSA level range: 0.14–24.3 ng/mL) who underwent a 3-T MRI examination followed by sextant TRUS-guided biopsy. For predicting recurrent cancer, combined T2WI and DWI showed Se 62%, Spe 97%, PPV 91%, and NPV 81%, whereas T2WI alone displayed Se 25%, Spe 92%, PPV 57%, and NPV 74%. A significantly greater *A*_*z*_ was found for combined T2WI and DWI (*A*_*z*_ = 0.879) as compared to T2WI alone (*A*_*z*_ = 0.612).

Morgan and colleagues [23] found that ADC has a high Se and good Spe for detecting local tumor recurrence after EBRT larger than 0.4 cm^2^. They enrolled 24 patients with rising PSA levels after 30–130 months EBRT. TRUS-guided biopsy was used as reference standard. The Se, Spe, PPV, and NPV of DWI for detecting recurrent tumor were 93.8%, 75%, 88.2%, and 85.7%, respectively. ROC analysis indicated that a cutoff ADC of 1216 × 10^−6^ mm^2^/s would differentiate tumor from nontumor irradiated peripheral zone and central gland with 100% Se and 96% Spe (*A*_*z*_, 0.992).

Hara et al. [24] in order to evaluate DWI for the diagnosis and localization of recurrent PCa after definitive EBRT retrospectively analyzed 10 patients with BF (PSA range: 2.06–7.36 ng/mL) using histological findings from 22-core 3-dimensional prostate mapping biopsy as a standard reference. On a patient-by-patient basis, Se and Spe were both 100%. On a region-by-region basis, they found a Se, Spe, PPV, NPV, and accuracy of 69%, 91%, 37%, 97%, and 89%, respectively.

The results of the aforementioned studies support that DWI may be considered as a useful functional technique to detect and localize PCa recurrence in patients with BF after EBRT and determine a target area for focal salvage therapy. DWI would also serve as a guide for targeted biopsy in order to improve the Se of TRUS-guided biopsy. On the other hand, DWI could be less useful in detecting local recurrences after ^125^I permanent prostate seed implants because seed implants in the prostate may cause artifacts, thus limiting the diagnostic accuracy of DWI.

Other studies compared DWI with the other functional techniques. Arumainayagam et al. [25] assessed the role of mp-MRI in evaluating post-RT local PCa recurrence, using transperineal template-guided 5 mm-spaced biopsies as a reference standard. They evaluated 13 men with BF after EBRT. The MR scan protocol included T1 and T2W sequences and DCE and DWI. They assessed the accuracy of mp-MRI without determining the relative contribution of each functional technique. Overall accuracy for reader 1 and 2, as expressed by the *A*_*z*_, was 0.77 and 0.89 for all cancer, with accuracies of 0.86 and 0.93 for those cancers with ≥3 mm biopsy core length.

Westphalen et al. [26] found that the incorporation of MRSI and/or DWI into T2WI significantly improves the assessment of patients with suspected recurrence after EBRT and a combined approach with all 3 modalities may have the best diagnostic performance. They reviewed 26 patients with BF. TRUS-guided biopsy was the standard of reference. The highest *A*_*z*_ in the detection of recurrence was found for the combination of T2WI plus DWI plus MRSI (0.869) followed by, in decreasing order, DWI plus MRSI (0.863), T2WI plus MRSI (0.841), T2WI plus DWI (0.774), MRSI alone (0.830), DWI alone (0.755), and T2WI alone (0.616). Even though MRSI appears to achieve good results by itself (the *A*_*z*_s for the combination of any two or all three MR techniques were not larger than that of MRSI alone), the combination of 3 modalities led to better results.

Kim and colleagues [27] showed that the use of combined DCEI and DWI may be more useful than the use of either DCEI or DWI alone for the prediction of locally recurrent cancer after definitive EBRT. They enrolled 24 patients with BF who underwent a 3-T MRI examination followed by TRUS-guided biopsy (PSA range: 0.14–11.2 ng/mL). In predicting locally recurrent cancer the Se and Spe were significantly higher for DWI (49% and 93%), DCEI (49% and 92%), and combined DCEI and DWI (59% and 91%) than for T2WI (27% and 80%). The accuracy of DWI (82%), DCEI (81%), and combined DCEI and DWI (83%) was greater than that of T2WI (67%). Moreover a significantly greater *A*_*z*_ was determined for combined DCEI and DWI (*A*_*z*_ = 0.863), as compared with T2WI, DCEI, and DWI alone (0.594, 0.737, and 0.782, resp.).

Akin et al. [28] found that in detecting local recurrence after RT, T2WI with DWI and DCEI yielded significantly higher diagnostic accuracy than T2WI alone. They enrolled 24 patients with BR after EBRT or permanent interstitial implantation (PSA level range: 0.43–6.3 ng/mL). TRUS-guided biopsy (12–16 cores) was used to validate MRI results. They found that when DWI and DCE images were added to T2WI, the accuracy in the detection of local recurrence at the patient and prostate-side levels increased significantly for both an experienced reader and an inexperienced reader. At the patient level, the *A*_*z*_ for reader 1 increased from 0.64 with T2WI alone to 0.95 with mp-MRI, and the *A*_*z*_ for reader 2 increased from 0.53 with T2WI alone to 0.86 with mp-MRI. At the prostate-side level, the *A*_*z*_ for reader 1 increased from 0.73 with T2WI alone to 0.90 with mp-MRI, and the *A*_*z*_ for reader 2 increased from 0.66 with T2WI alone to 0.79 with mp-MRI. In addition, interreader agreement was higher for the interpretation of mp-MRI than for the interpretation of T2WI alone, with the weighted *K* statistic increasing from 0.38 to 0.79 at the patient level and 0.32 to 0.61 at the prostate-side level. The limitations of this study include its retrospective design, small sample size, and TRUS-guided biopsy used as the reference standard.

In a recent study Donati and coworkers [29] analyzed a patient population of 53 men with BR after EBRT. TRUS-guided biopsy was used as the standard of reference. They showed that in detecting recurrent tumour combined T2WI and DWI provide significantly better diagnostic accuracy (*A*_*z*_ of 0.79–0.86 for reader 1 and 0.75–0.81 for reader 2) than of T2WI alone (*A*_*z*_ of 0.63–0.67 for reader 1 and 0.46–0.49 for reader 2) and that the addition of DCEI to T2WI and DWI did not improve diagnostic accuracy in the detection of locally recurrent PCa after RT. Moreover T2WI with DWI also yielded higher interreader agreement than any other combination tested (*k* = 0.17–0.20 for T2WI alone, *k* = 0.55–0.63 for T2W and DWI, *k* = 0.32–0.34 for T2W and DCE, and *k* = 0.49–0.58 for T2WI, DWI, and DCEI). Therefore according to these data, in the detection of locally recurrent PCa in patients who underwent RT, the combination of T2WI with DWI appears to be the optimal approach, as the further addition of DCE sequences to this combination did not yield any incremental value, thus eliminating the risks and costs associated with the intravenous administration of gadolinium-based contrast agents. However, this study has several limitations such as (1) the retrospective analysis of patients who presented over more than 3 years; thus variations in imaging protocols (different *b* values for DWI or temporal resolution for DCEI) may have affected imaging results, (2) the use of TRUS-guided biopsy and not step-section pathologic analysis of prostatectomy specimens as the reference standard, and (3) MRSI was not included in mp-MRI protocol. Moreover, Donati et al. attempted to assess the correlation between quantitative mp-MRI-derived parameters (ADC from DWI, *K* ^trans⁡^ and *k*_ep_ from DCEI) and the Gleason score. Although the Gleason score has been shown to correlate with quantitative mp-MRI parameters in the untreated prostate [101–103], they did not find a significant association between the Gleason score and these parameters in irradiated prostates. The authors hypothesized that the lack of association between mp-MRI parameters and aggressiveness may partly be due to the reference standard used or the small sample size.

Roy et al. [11] in a recent study evaluated the Se in the detection of post-RT local PCa recurrence of the 3 types of functional MRI techniques, with TRUS-guided biopsy as the standard of reference. They enrolled 32 patients with BR after EBRT (PSA serum level range: 2.2–4.8 ng/mL). The Se was highest for T2WI plus DWI plus DCEI plus MRSI (100%) followed by, in decreasing order, T2WI plus DWI, DCEI alone and DWI alone (94%), T2WI plus DCEI (91%), T2WI plus DWI plus DCEI plus MRSI (76%), T2WI and MRSI alone (74%), and lastly T2WI plus MRSI (44%).

## 7. Final Considerations

According to Beresford et al. [104], whenever BF is observed after RP, the current practice is to treat patients with salvage therapy once metastatic disease has been excluded, without the need for imaging or histological evidence of local recurrence, accepting that current techniques may not be sensitive enough to detect small volume local disease at low PSA values and slow PSA kinetics. Against this assumption, an increasing number of studies have demonstrated that mp-MRI is a very useful tool in confirming the diagnosis of local PCa recurrence after RP. It is indicated to diagnose small local cancer recurrence in a range of PSA serum values between 0.2 and 1 ng/mL when PET/CT is not eligible. Moreover Mp-MRI, thanks to functional techniques, allows the differentiation between residual glandular healthy tissue, scar/fibrotic tissue, granulation tissue, and tumour recurrence and it may also be able to assess the aggressiveness of nodule recurrence. Moreover, the recent development of hybrid PET/MRI scanners could improve the diagnostic accuracy in depicting local PCa relapses in post-prostatectomy fossa. Mp-MRI findings could be used to boost the dose of salvage RT to the recurrent PCa nodule and potentially improve the control of local disease, thus avoiding an eventual locoregional relapses.

In current clinical practice, when a local PCa recurrence after definitive RT is diagnosed, the most popular treatment strategy is still some form of whole-gland salvage therapy because the exact location of the recurrent tumor within the prostate is generally unknown. At present, mp-MRI is widely considered to be the state of the art in detecting and localizing, in a timely manner, PCa recurrence in patients with BP after definitive RT. The precise detection and localization of local tumor recurrence are of utmost importance for several purposes: (1) for guiding targeted TRUS-guided biopsy of suspicious areas, thus reducing the false-negative rate associated with systematic biopsies, (2) to perform MR-guided biopsy leading to a higher detection rate of recurrent PCa with a minimum number of biopsy cores compared to TRUS-guided systemic biopsies and, consequently, leading to higher patient satisfaction, (3) appropriate treatment selection and planning, (4) to guide surgery, and (5) to improve the targeting of salvage RT (external beam or interstitial) or minimally invasive ablative techniques in order to perform focal salvage therapies effectively with minimal complications. Another advantage of mp-MRI is its potential to change patient management. For instance, in patients with BR after RT, who are considered for salvage prostatectomy, MRI findings of SV invasion or extraprostatic extension will alter patient management.

To minimize time for mp-MRI data acquisition, we hypothesize that a combination of T2WI and DWI could be sufficient to detect local recurrence. This observation derives from the evidence that DWI and DCEI are similar in terms of Se and accuracy for recurrent PCa detection and localisation both after RP and EBRT [10, 29]. Therefore, since DWI requires a short imaging time, without the need for intravenous contrast medium and has relatively simple post-processing requirements, it could be assumed to be a valid alternative to DCE. However, DCEI can be helpful in patients with seed placement after BT, as DWI is prone to susceptibility artifacts and distortion in these cases. As far as MRSI is concerned, even if it has shown acceptable accuracy and diagnostic performance, its disadvantages are a longer acquisition time and the need for additional software, which leads to increased costs and decreased throughput. Moreover, MRSI is technically challenging after BT, because the numerous implanted metallic seeds affect the local magnetic field homogeneity, yield susceptibility artifacts, and decrease the SNR, thus impairing the quality of the spectra. Because of these drawbacks, we can postulate that, in the daily work flow, this sequence is not necessary and must not be included in the routine protocol.

In conclusion, mp-MRI could be currently considered as the most reliable imaging biomarker to detect local PCa recurrence in patients with BF after RP or definitive RT; the major advantage could be achieved in presence of low rising PSA level, in order to perform an early and relevant treatment.

## Figures and Tables

**Table 1:** Characteristics of the different reviewed studies on mp-MRI for the diagnosis of local recurrence after RP.

Authors	MRI scan	Study design	Cases	Mean PSA	Mean lesion size	Reference standard	T2WI	DCE	MRSI	DWI	Combined techniques
Silverman and Kerbs [3]	ERC 1.5-T	Prospective	41	1.4 ng/dL	16 mm	TRUS biopsy	Se 100% Spe 100% PPV 100% NPV 100%				

Sella et al. [4]	PAC + ERC 1.5-T	Retrospective	48	2.18 ng/dL	14 mm	TRUS biopsy, PSA reduction after RT, increase lesion size at MRI	Se 95% Spe 100%				

Cirillo et al. [5]	PAC + ERC 1.5-T	Retrospective	72	1.51 ng/dL	17 mm	TRUS biopsy, Cho-PET findings, PSA reduction after RT	Se 61.4% Sp 82.1% PPV 84.4% NPV 57.5% Acc 69.4%	Se 84.1% Spe 89.3% PPV 92.5% NPV 78.1% Acc 86.1%			

Casciani et al. [6]	PAC + ERC 1.5-T	Retrospective	46	1.9 ng/dL	15 mm	TRUS biopsy, PSA reduction after RT	Se 48% Sp 52% PPV 54% NPV 46% Acc 48%	Se 88% Spe 100% PPV 100% NPV 88% Acc 94%			

Sciarra et al. [7]	PAC + ERC 1.5-T	Prospective	70	1.2 ng/dL (group A) 0.8 ng/dL (group B)	13 mm (group A) 6 mm (group B)	TRUS biopsy (group A) PSA reduction after RT (group B)		Se 71–79% Spe 94–100% PPV 96–100% NPV 67–79% *A*_*z*_ 0.923–0.931	Se 71–84% Spe 83–88% PPV 91–93% NPV 56–74% *A*_*z*_ 0.81–0.942		MRSI + DCE: Se 86-87% Spe 94–100% PPV 96–100% NPV 75–79% *A*_*z*_ 0.964–0.94

Panebianco et al. [8]	PAC + ERC 3.0-T	Prospective	84	1.1 ng/dL (group A) 1.9 ng/dL (group B)	6 mm (group A) 13 mm (group B)	TRUS biopsy (group B) PSA reduction after RT (group A)					MRSI + DCE: Se 92–94% Spe 75–100% PPV 96–100% NPV 57–60% Acc 89–94% *A*_*z*_ 0.833–0.971

Wu et al. [9]		Meta-analysis					Se 72% Spe 74%	Se 85% Spe 95%			MRSI + DCE: Se 92% Spe 95%

Panebianco et al. [10]	PAC + ERC 3.0-T	Prospective	262	1.3 ng/dL (group A) 2.0 ng/dL (group B)	5 mm (group A) 12 mm (group B)	TRUS biopsy (group A) PSA reduction after RT (group B)					T2WI + DCE: Se 98–100% Spe 94–97% PPV 96-97% NPV 95-96% Acc 91% *A*_*z*_ 0.875–0.917 T2WI + DWI Se 97-98% Spe 95-96% PPV 93–96% NPV 91–95% Acc 89–92% *A*_*z*_ 0.783–0.823

Roy et al. [11]	PAC + ERC 3.0-T	Retrospective	28	0.98 ng/dL		TRUS biopsy	Se 56%	Se 94%	Se 50%	Se 65%	T2WI + DCE: Se 97% T2WI + DWI + DCE: Se 94% T2WI + DWI + DCE + MRS: Se 74% T2WI + DWI: Se 65% T2WI + MRS: 53%

PAC: phased-array coil; ERC: endorectal coil; Se: sensitivity; Spe: specificity; PPV: positive predictive value; NPV: negative predictive value; Acc: accuracy; *A*_*z*_: area under the receiver operating characteristic curve.

**Table 2:** Characteristics of the different reviewed studies on mp-MRI for the diagnosis of local recurrence after RT.

Authors	MRI scan	Study design	Cases	Mean PSA	Reference standard	T2WI	DCE	MRSI	DWI	Combined techniques
Sala et al. [12]	PAC + ERC 1.5-T	Prospective	45	3.57 ng/dL	Whole-mount RP section	Se 36–76% Spe 65–81% *A*_*z*_ 0.61–0.75				

Westphalen et al. [13]	PAC + ERC 1.5-T	Retrospective	59		TRUS biopsy	Se 62–74% Sp 64–68% PPV 70–80% NPV 50–70% Acc 63–71%				

Rouvière et al. [14]	PAC 1.5-T	Prospective	22	6.36 ng/dL	TRUS biopsy	Se 26–44% Sp 64–86% PPV 47–63% NPV 61–69% Acc 54–60%	Se 70–74% Sp 73–85% PPV 68–78% NPV 78-79% Acc 73–79%			

Haider et al. [15]	PAC 1.5-T	Prospective	33	2.1 ng/dL	TRUS biopsy	Se 38% Sp 80% PPV 24% NPV 88% Acc 74%	Se 72% Sp 85% PPV 46% NPV 95% Acc 83%			

Kara et al. [16]	PAC 1.5-T	Retrospective	20		TRUS biopsy	Se 86.7% Sp 100% PPV 100% NPV 71% Acc 90%	Se 93.3% Sp 100% PPV 100% NPV 83.3% Acc 95%			

Menard et al. [17]		Prospective	35		TRUS biopsy			Se 88.9% Sp 92% PPV 91.7% Acc 91.4%		

Coakley et al. [18]	PAC + ERC 1.5-T	Retrospective	21	2.3 ng/dL	TRUS biopsy	*A*_*z*_ 0.49–0.51		Se 89% Sp 82% PPV 64% NPV 96% *A*_*z*_ 0.81		

Pucar et al. [19]	PAC + ERC 1.5-T	Prospective	9	3.7 ng/dL	Whole-mount RP section	Se 68% Sp 96%		Se 77% Sp 78%		

Westphalen et al. [20]	PAC + ERC 1.5-T	Retrospective	64	2.6 ng/dL	TRUS biopsy	*A*_*z*_ 0.67		T2WI + MRS: *A*_*z*_ 0.79		

Wu et al. [9]		Metanalysis				Se 74% Spe 65%	Se 90% Spe 81%			MRSI + DCE: Se 90% Spe 90%

Tamada et al. [21]	PAC 1.5-T	Retrospective	16	7.42 ng/dL	TRUS biopsy	Se 27% Sp 99% PPV 86% NPV 87% Acc 87%	Se 50% Sp 98% PPV 85% NPV 90% Acc 90%		Se 68% Sp 95% PPV 75% NPV 94% Acc 91%	T2WI + DCE + DWI: Se 77% Sp 92% PPV 68% NPV 95% Acc 90%

Kim et al. [22]	PAC 3.0-T	Prospective	36	3.44 ng/dL	TRUS biopsy	Se 25% Sp 92% PPV 57% NPV 74% *A*_*z*_ 0.612				T2WI + DWI: Se 62% Sp 97% PPV 91% NPV 81% *A*_*z*_ 0.879

Morgan et al. [23]	ERC 1.5-T	Retrospective	24		TRUS biopsy				Se 93.8% Sp 75% PPV 8.2% NPV 85.7%	

Hara et al. [24]	PAC 1.5-T	Retrospective	10	4.44 ng/dL	TRUS biopsy				Se 69% Sp 91% PPV 37% NPV 97% Acc 89%	

Arumainayagam et al. [25]	PAC 1.5-T	Retrospective	13		TRUS biopsy					T2WI + DCE + DWI: *A*_*z*_ 0.86–0.93

Westphalen et al. [26]	PAC + ERC 3.0-T	Retrospective	26	2.5 ng/dL	TRUS biopsy	*A*_*z*_ 0.616		*A*_*z*_ 0.830	*A*_*z*_ 0.755	T2WI + MRS + DWI: *A*_*z*_ 0.869 MRS + DWI: *A*_*z*_ 0.863 T2WI + MRS: *A*_*z*_ 0.841 T2WI + DWI: *A*_*z*_ 0.774

Kim et al. [27]	PAC 3.0-T	Retrospective	24	2.76 ng/dL	TRUS biopsy	Se 27% Sp 80% PPV 32% NPV 76% Acc 67% *A*_*z*_ 0.594	Se 49% Sp 92% PPV 67% NPV 84% Acc 81% *A*_*z*_ 0.737		Se 49% Sp 93% PPV 72% NPV 84% Acc 82% *A*_*z*_ 0.782	DCE + DWI: Se 59% Sp 91% PPV 69% NPV 87% Acc 83% *A*_*z*_ 0.863

Akin et al. [28]	PAC + ERC 1.5-T	Retrospective	24	1.63 ng/dL	TRUS biopsy	Se 13–81% Sp 25–88% *A*_*z*_ 0.53–0.64				T2WI + DCE + DWI: Se 81–94% Sp 75–100% *A*_*z*_ 0.86–0.95

Donati et al. [29]	PAC + ERC 1.5 or 3.0-T	Retrospective	53		TRUS biopsy	Se 54–66% Sp 39–61% PPV 63–77% NPV 30–48% *A*_*z*_ 0.46–0.632				T2WI + DCE: Se 60–63% Sp 56–89% PPV 73–91% NPV 44–53% *A*_*z*_ 0.601–0.830 T2WI + DWI: Se 54–83% Sp 89–94% PPV 93–95% NPV 52–73% *A*_*z*_ 0.812–0.863 T2WI + DCE + DWI: Se 49–71% Sp 94% PPV 94–96% NPV 49–63% *A*_*z*_ 0.722–0.879

Roy et al. [11]	PAC + ERC 3.0-T	Retrospective	32	3.6 ng/dL	TRUS biopsy	Se 74%	Se 94%	Se 74%	Se 94%	T2WI + DCE: Se 91% T2WI + DWI + DCE: Se 100% T2WI + DWI + DCE + MRS: Se 76% T2WI + DWI: Se 94% T2WI + MRS: 44%

PAC: phased-array coil; ERC: endorectal coil; Se: sensitivity; Spe: specificity; PPV: positive predictive value; NPV: negative predictive value; Acc: accuracy; *A*_*z*_: area under the receiver operating curve.
